# A propagation-based seed-centric local community detection for multilayer environment: The case study of colon adenocarcinoma

**DOI:** 10.1371/journal.pone.0255718

**Published:** 2021-08-09

**Authors:** Ehsan Pournoor, Zaynab Mousavian, Abbas Nowzari-Dalini, Ali Masoudi-Nejad

**Affiliations:** 1 Laboratory of Systems Biology and Bioinformatics (LBB), Institute of Biochemistry and Biophysics, University of Tehran, Tehran, Iran; 2 School of Mathematics, Statistics, and Computer Science, College of Science, University of Tehran, Tehran, Iran; Universiteit Maastricht, NETHERLANDS

## Abstract

Regardless of all efforts on community discovery algorithms, it is still an open and challenging subject in network science. Recognizing communities in a multilayer network, where there are several layers (types) of connections, is even more complicated. Here, we concentrated on a specific type of communities called seed-centric local communities in the multilayer environment and developed a novel method based on the information cascade concept, called PLCDM. Our simulations on three datasets (real and artificial) signify that the suggested method outstrips two known earlier seed-centric local methods. Additionally, we compared it with other global multilayer and single-layer methods. Eventually, we applied our method on a biological two-layer network of Colon Adenocarcinoma (COAD), reconstructed from transcriptomic and post-transcriptomic datasets, and assessed the output modules. The functional enrichment consequences infer that the modules of interest hold biomolecules involved in the pathways associated with the carcinogenesis.

## Introduction

In the network analysis fields, such as social networks and network biology, community detection is an essential topic in discovering strongly interconnected objects with similar identity or behavior [1, 2]. The problem of unfolding community structures is known to be computationally difficult to solve, while its approximate solutions have to cope with accuracy and efficiency issues that become more severe when the network gets larger [3]. It was proven that these communities (modules) play vital roles in the performance of the whole system and their identification yields remarkable empirical insights about complex systems [4–8]. For example, in a metabolic network, communities correspond to the biological functions of the cell [9, 10]. In the same way, in a web graph, communities exhibit topics of interest [11, 12]. Presently, there are varieties of methods to explore communities in networks with dissimilar topological properties [13–16]. In this context, various graph community detection algorithms have been published [2, 14, 17–19] which were primarily categorized in two classes of agglomerative and divisive. Over time, different approaches, such as overlapping vs. non-overlapping [20–22], local vs. global [23–26], sparse vs. dense [14, 27, 28], and single-layer vs. multilayer [25, 29–32] community detection methods were defined that their difference is in their underlying method, application or use case datasets.

The introduction and formulation of multilayer networks [33–35], in which every layer signifies a distinct variety of interaction between nodes, has attended more interest of graph mining researchers [29–32, 36, 37]. There is a consensus among academics that identifying communities in a single network is not sufficient to analyze the structure and the performance of a real-world system [38–40]. However, the complex foundation of multilayer networks makes it challenging to explore communities precisely. In the context of multilayer community detection, layer-aggregation, ensemble clustering, and multilayer-extension are three methodologies used by publications [41]. It has been shown that the multilayer-extended approach (simultaneous analysis) overtakes the layer-aggregated or ensemble solutions [42, 43]. There are several recent multilayer-extended techniques [29, 44, 45], which are mainly designed for the global community detection problem. Now, despite these prominent methods, it is an open and hot topic to optimize and improve those algorithms.

Multilayer community detection is a general term that may be *Global* or *Local*. In global community detection, the goal is to explore all communities inside the whole network. However, local community identification is a distinct and beneficial category in which the objective is to explore the community surrounding a predefined node, called seed [46]. This is helpful when there exists a known seed and we are seeking for the other interrelated and analogous nodes [23, 41, 46–49]. For example, when we are looking for all friendship communities in a school dataset, we are performing global community detection. Whereas, when we look for the friendship community of a special person, we explore his/her local community. It is noteworthy that, a local method could be used as global by seeding different nodes. However, choosing the correct set of seeds will be effective in this process [46–48]. In this case, multiple seeds may correspond to the same community. Some seed-based global approaches, first, specify seed nodes (such as the hub or central nodes), then predict their surrounding communities. In the school friendship example, this is equivalent to selecting several people, then finding their local communities. Various publications on seed-based graph partitioning subject reveal its importance in real-world applications [20, 21, 23, 24, 47].

Recently, the application of the multilayer approach in diverse regions of natural science has opened a new outlook to explore world facts. In biology, layers are reconstructed from genomics, transcriptomics, proteomics, metabolomics, and signaling data, and analyzed computationally to realize reasons for biological events such as disorders [50–56]. Herein, we offered a new seed-centric community detection algorithm based on the network propagation theory, named “Propagation-based Local Community Detection for Multilayer environment” (PLCDM). In this method, the community is discriminated against based on the information flow extent of its containing nodes. An information cascade from the seed node will target its neighborhood, especially neighbors with tight connections. We utilized this idea to identify the most affected zone around the seed node as its surrounding module. To mimic such a propagation strategy, a biased random walk with a random restart process is employed. The process of information cascade (implemented by random walk) from the seed object is repeated several times and the module containing more portion of the information is selected as the community structure for that seed. The proposed technique is tested on one real and two simulated datasets and the obtained results demonstrate that modules specified in this way are more accurate than former methods with a small false discovery rate. The source code of PLCDM implementation is available on our GitHub page (https://github.com/LBBSoft/PLCDM).

**Fig 1:**
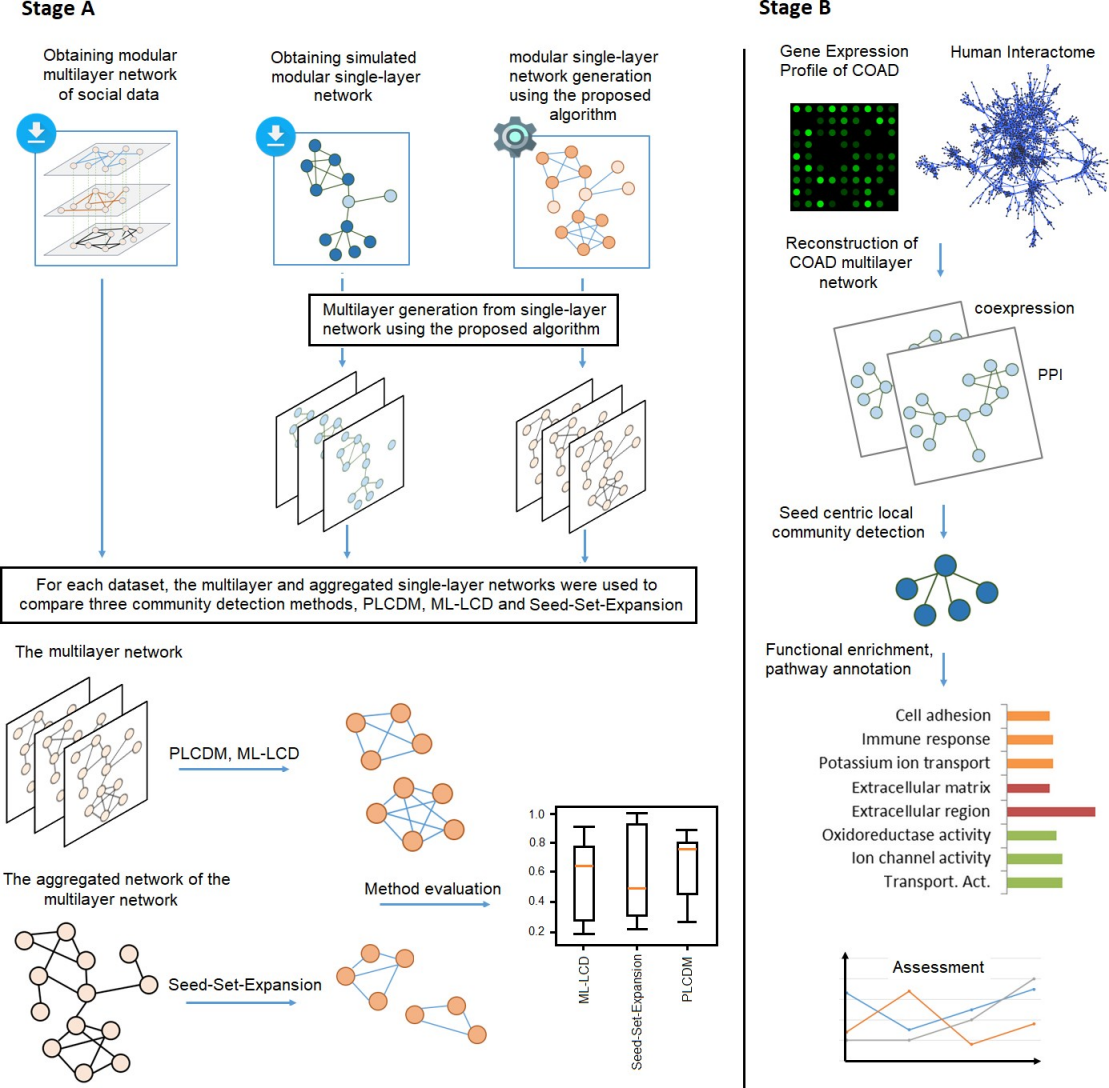
The roadmap of the study. **Stage A)**, Method evaluation. Here, to verify the performance of the proposed algorithm, three datasets were used: 1- a multilayer network constructed from a generated artificial modular single-network (created through a modular single-network generation algorithm, see S1 Appendix), 2- a multilayer network simulated from a synthetic modular single-network provided by the Graph Challenge data repository, and 3- a real social multilayer network published by Rossi et al. [57], based on five social interconnections (Facebook, lunch, coauthor, leisure, and work). The multilayer simulation algorithm in cases 1 and 2 was described in S1 Appendix. In all these three multilayer datasets, gold-standard communities were specified previously. For every dataset, the original multilayer and the aggregated single-layer network is used by methods PLCDM and ML-LCD (multilayer-specific), and Seed-Set-Expansion (single-layer-specific) to explore communities. **Stage B),** Application of the PLCDM on a reconstructed two-layer network of the colon adenocarcinoma (COAD).

In the roadmap of this study, as depicted in Fig 1, two stages of implementation were defined: first, evaluation of the PLCDM, and second, a case study of the application of PLCDM on the colon cancer data to identify related biomolecules involved in carcinogenesis. Besides, in the first stage, to prepare gold-standard data for the verification of the suggested methodology, we designed two additional algorithms: 1- modular graph generation, and 2- multilayer network simulation from a single-layer graph, which are both reported in the Supporting information.

## Materials and methods

### Community detection

Herein, we presented a multilayer seed-based local community detection approach based on a biased random walk with a random restart process, simulating information diffusion from a predefined seed node. From this viewpoint, in a multilayer graph *G* (Eq 1) with *n* = |*V*| nodes and *k* = |*L*| layers, *L* = {*l*_1_, *l*_2_,…,*l*_*k*_}, indicating different types of interactions (undirected networks), a local community around the seed node *s* is defined as a set of nodes having a considerable extent of information streamed from *s* and shared by layers.


$$G=<V,E_{1},E_{2},\ldots ,E_{k}:\;E_{x}\subseteq V\times V,\forall \;x\;\epsilon \;\{1,\ldots ,k\}>$$ (1)

To achieve such a community, utilizing the information diffusion idea, we established a graph random walker that moves randomly to neighbor nodes and scores them in each step. Then the community is exploited based on the scores that each node gained. To implement this kind of random walk, we have considered three criteria that are formulated in Eqs 2 to 5. First, we predisposed the walker to move to neighbors that have a large number of neighbors in common with the primary node. It originates from social relationships that the greater the number of mutual friends between two individuals, the stronger friendship (higher diffusion probability) between them. If we were to describe this phenomenon in a biological subject as protein-protein interaction (PPI) network, the two receptors that bind to a large number of common ligands are similar in 3-D structure and conformation and have analogous binding sites. Therefore, they may have the same function and activity and are likely to be in the same biological pathway (module). Second, the walker should not go in the direction of nodes of other communities that have common neighbors with the host node. Without this constraint, in a scale-free topology, the walker tends to move toward other hub nodes (which have more common neighbors and their degree is high). In another biological simulation, this constraint prevents housekeeping genes from being part of a particular pathway (community). Third, selecting common connections is another critical point that leads to a correct calculation. For example, if there are different relationships between two people (Facebook, Twitter, email, etc.), there will be a high probability that they are both in the same community. Alternatively, in a biological example, if there is a co-expressional pattern between the two genes and their products form protein complexes, this increases the likelihood of their joint involvement in biological processes. This tendency should be reflected in the behavior of random walker. To this end, we let the walker move through edges that exist in most layers with higher probability. Applying common-edges influence in the random walk probability will lead to the extraction of a shared structure between the layers. We multiply this value to the normalized weight to force the walker to move towards the common structure between layers.

Mathematically, we formulated these three concerns in Eq 2, where *P*_*l*_(*i*,*j*) is the intra-layer probability of moving from a node *i* to a node *j* in layer *l*. In this equation, each term considers one of the constraints mentioned above. It is worth mentioning that Γ_*l*_(*i*) denotes the neighbor set of node *i* in layer *l* (Eq 3), and *ω*(*i*,*j*) is the weight of edge (*i*,*j*) (defined in Eq 4) in the multilayer network; However, other edge weighting methods could be utilized (i.e. Entropy-based weighting proposed by Hmimida et al. [41]). It is a value in the range [0, 1] and does not depend on the layer; it is used to smooth probabilities. Also, *A*_*l*_ stands for the adjacency matrix of layer *l*.


$$P_{l}(i,j)=\frac{|\Gamma_{l}(i)\cap \Gamma_{l}(j)|+1}{|\Gamma_{l}(i)|+1}\times \frac{1}{{|\Gamma }_{l}(j)|+1}\times \omega (i,j)$$ (2)

$$\Gamma_{l}(i)=\{\forall \;j\epsilon \;V:(i,j)\in E_{l}\}$$ (3)

$$\omega (i,j)=\frac{\sum_{l}A_{l}(i,j)}{k},\;where\;A_{l}(i,j)\;\epsilon \;\{0,1\}$$ (4)

**Fig 2:**
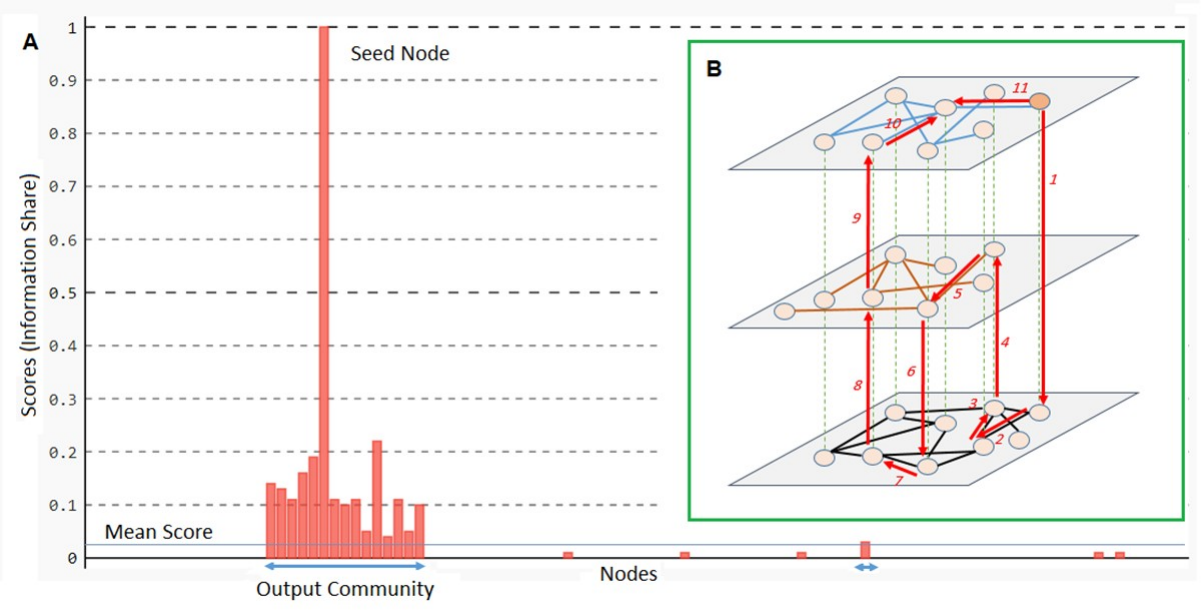
Demo of community detection procedure. **(A)** Extraction of a community using the information share (z-score) extent of vertices. **(B)** Random walk with random restart process from a seed node in a typical multilayer network.

In addition, the random walker must be capable to change layers and score nodes according to the topology of layers. Here in Eq 5, *R*_*i*_(*l*_*s*_, *l*_*t*_) denotes the probability of moving from a typical node *i* in layer *l*_*s*_, to its counterpart node *i* in layer *l*_*t*_. Ideally, as much as the two counterpart nodes are similar in their neighbors, the probability of moving between them should be larger. To capture this kind of similarity, we used the *Jaccard similarity* measure. Here also, it is possible that in a layer, a node to be isolated without any connection to other nodes. Therefore, to prevent division by zero we added 1 to the numerator and denominator of the equation. A typical information diffusion process (simulated by a random walk with random restart from a seed node) in the multilayer network is illustrated in Fig 2B.


$$R_{i}(l_{s},l_{t})=\frac{|\Gamma_{l_{s}}(i)\cap \Gamma_{l_{t}}(i)|+1}{|\Gamma_{l_{s}}(i)\cup \Gamma_{l_{t}}(i)|+1}$$ (5)

Starting from the seed node and proceeding random walks toward the most probable neighbors, nodes in the vicinity of the seed node will have a score indicating their share of information. After a few steps (which is specified randomly), the walker jumps to the seed node to start a new propagation. This is equal to multiple parallel information cascade from the host node *s*.

It is worth mentioning that PLCDM is applicable to single-layer graphs (e.g., aggregation of multilayer networks). In this condition, the walker will always move on the existing layer without changing layers.

Algorithm 1. *PLCDM*

*1*.      ***Input*:** *multilayer*, *seed*, *iteration_count*, *random_jump_prob*, *layer_change_prob*

*2*.      ***Output*:** *community*

*3*.      *V* = set of nodes in the *multilayer*

*4*.      *n* = number of nodes

*5*.      *layers* = set of layers in the *multilayer*

*6*.      *k* = number of layers

*7*.      *scores* = vector of nodes scores initialized to 0

*8*.      *R* = inter-layer transition probability matrices initialized to *1/k*

*9*.      *P* = intra-layer transition probability matrices initialized to *1/n*

*10*.      for layer *l* in layers:

*11*.         for nodes *i*, *j* in *V*:

*12*.            calculate *P*_*l*_(*i*,*j*)

*13*.      for node *i* in *V*:

*14*.         for layers *s*, *t* in *layers*:

*15*.            calculate *R*_*i*_(*l*_*s*_,*l*_*t*_)

*16*.      *current_node* = *seed*

*17*.      *scores[current_node] + = 1*

*18*.      *current_layer* = choose a layer randomly from layers

*19*.      for 1 → *iteration_count*:

*20*.         *neighbors* = get neighbors of *current_node*

*21*.         *current_node* = select a neighbor randomly from *neighbors* considering intra-layer probabilities *P*

*22*.         *scores[current_node] + = 1*

*23*.         *jump* = jump_or_stay(*random_jump_prob*)

*24*.         if *jump*:

*25*.            *current_node = seed*

*26*.            *scores[current_node] + = 1*

*27*.         *change_layer* = change_layer_or_stay(*layer_change_prob*)

*28*.         If *change_layer*:

*29*.            *current_layer* = select a layer randomly from *layers* considering inter-layer probabilities *R*

*30*.      *scores* = normalize (*scores*)

31.      *community* = select nodes with score > 0

*32*.      return *community*

As the scores are calculated, they are transformed by the simple z-score method [58]. With this transformation, not only scores fall in a smaller range, but also, scores larger than the average will be positive, and scores smaller than the average will be negative. Finally, the output community is specified from nodes that their normalized score is positive (their scores are larger than the average, see Fig 2A). At every step, probabilities of moving to the neighbors of the current node are rescaled such that their summation is one. The whole procedure is described in Algorithm 1.

The proposed method is also applicable to single-layer graphs. In this case, there is not any layer change (the *change_layer_or_stay* function in the algorithm always will return *false*), and the walker keeps moving based on intra-layer probabilities.

The algorithmic complexity of PLCDM is composed of the complexity of inter-layer and intra-layer probabilities calculation, and the random walk iterations. By considering *n* as the number of nodes in the multilayer network, and *k* as the number of layers such that *n*≫*k*, the total complexity is [*O*(*kn*^2^)+*O*(*nk*^2^)+*O*(*t*)]≅*O*(*kn*^2^) where *t* is the number movements (a constant value). Here, *O*(*kn*^2^) is the complexity of intra-layer probabilities calculations and *O*(*nk*^2^) denotes the complexity of inter-layer probabilities calculations. It demonstrates that the time-consuming part is the process of calculating intra-layer probabilities of layers, and then the output community is extracted in a linear time. Moreover, the memory space complexity is [*O*(*kn*^2^)+*O*(*nk*^2^)+*O*(*n*)]≅*O*(*kn*^2^). For example, in a typical implementation of the algorithm for a 5-layer network and 10^4^ nodes in each layer, it uses about 3.73 GB of memory and runs in 23 minutes to calculate the probability matrices. Then, the calculation of the output community will terminate in a few seconds. However, the time consumed depends on the processing power of the system.

### Network preparation

For the two stages accomplished in this study, stages A and B in Fig 1, different datasets were utilized. To confirm the performance of the presented method (stage A), we need gold-standard multilayer networks with prespecified communities. Here, three datasets with predefined ground-truth communities were employed; one real multilayer network (based on five social interconnections: Facebook, lunch, coauthor, leisure, and work), labeled Multilayer1, published by Rossi et al. [57], and two simulated multilayer networks (S1 Appendix) from two distinct single-layer graphs, prepared by (1) Graph Challenge data repository (https://graphchallenge.mit.edu/data-sets), labeled Multilayer2, and (2) our modular network generation algorithm available in S1 Appendix, labeled Multilayer3. In the case of multilayer simulation from a single-layer network (see S1 Appendix), since the generation is achieved using a probabilistic model; it is obvious that the obtained network from the layer aggregation of the simulated multilayer network, differs from the original single-layer network. In all these three multilayer networks, edges are undirected and unweighted and nodes may be different in every layer.

At stage B, we apply the PLCDM method on a reconstructed two-layer network of colon cancer to test if it can uncover functional modules having a role in the carcinogenesis biological pathways. In this multilayer, the first layer is a gene co-expression, and the second one is a protein-protein interaction graph. In the co-expression layer, nodes are genes, and edges show similar expressional patterns between genes. However, in the PPI layer, nodes are proteins, and their interrelations are physical binding interactions. As proteins are gene products, and to align layer nodes, we used the corresponding gene symbols as node labels in both layers. This structure is used in multiple earlier works [36, 59, 60]. In the proposed structure, we only considered intra-layer and coupling inter-layer edges, regardless of possible (non-coupling) gene-protein binding interactions. Herein, two types of biological data were utilized to model cancer states. For the co-expression layer, FPKM-UQ (Fragments Per Kilobase of transcript per Million mapped reads upper quartile) normalized gene expression profile of COAD patients were downloaded from the GDC data portal (https://portal.gdc.cancer.gov/). After preprocessing the expression data, genes with the following conditions were excluded: (1) genes with missing values in any sample [61], (2) genes with the expression count value of zero in more than 80% of all samples [61–63], (3) genes that possessed the expression rates with zero standard deviation across all samples, and (4) genes with average CPM (count per million) lower than 1 [61]. Afterward, a gene co-expression network was reconstructed using the WGCNA package [64] in R. For the co-expression layer, we defined a correlation threshold to filter significant edges. Conversely, in the second layer, curated Protein-Protein interactions (PPIs) for the same genes were pulled out from interactome data published by Menche et al. [65]. Here there is an assumption: in the reconstruction of this multilayer network, if a gene encodes several proteins or vice versa, to simplify the problem, only one-to-one mappings are considered. However, implementing one-to-many mappings is possible by letting the walker to select randomly from correspondents in layer change process.

### Method evaluation

To assess the method truthfulness in finding correct modules, we compared PLCDM with two other prominent local community detection methods, ML-LCD [25] and Seed-Set-Expansion [21]. ML-LCD is a multilayer seed-centric local community detection and, on the other hand, Seed-Set-Expansion is a single-layer local community detection method. First, we compared PLCDM with ML-LCD, by applying both methods on every multilayer network of stage A, declared in the Network Preparation section. Second, we are interested in investigating PLCDM performance on the three multilayer networks, in comparison with the Seed-Set-Expansion performance on the aggregated networks of those multilayers. Our objective from performing this two-step evaluation is not only assessing PLCDM accuracy against other methods, but also its advantages over layer aggregation. In every multilayer network (one real and two synthetic networks), ground-truth communities were specified previously. Each node inside these predefined communities is used as a seed item, and the resulting community is calculated using PLCDM, ML-LCD, and Seed-Set-Expansion algorithms. Afterward, these three methods are compared to see the similarity of their result community to the ground-truth community.

For example, suppose that for a multilayer network M, the set of predefined ground-truth communities is ℂ = {*C*_1_, *C*_2_, *C*_3_} in which every community contains some nodes (e.g. *C*_1_ = {*v*_1_, *v*_2_, *v*_3_, *v*_4_, *v*_5_}). In the evaluation process, every node such as *v*_1_ is considered as the seed, and using the three methods (PLCDM, ML-LCD and Seed-Set-Expansion), their output communities are predicted (e.g. $O_{\left(PLCDM,v_{1}\right)}=\{v_{1},v_{2},v_{3},v_{6},v_{7}\},\;O_{\left(ML-LCD,v_{1}\right)}=\{v_{1},v_{3},v_{5},v_{7},v_{8}\}$), and then compared to the original ground-truth community *C*_1_. Based on these comparisons, values of contingency tables (*TN*, *TP*, *FN*, and *FP*) could be identified (*e*. *g*. *TP*_*PLCDM*_ = 3 and *FP*_*PLCDM*_ = 2). Utilizing these contingency tables, methods are examined by measures: *Specificity*, *Precision*, *Recall*, *Accuracy*, *F1*, *MCC (Matthews Correlation Coefficient)*, *FDR (False Discovery Rate)*, and *NMI (Normalized Mutual Information)* explained in Table 1. This practice is performed for entire nodes of ground-truth communities. Now, for every seed item of ground-truth communities, we have a set of evaluation measures that could be used to compare methods.

**Table 1:** Applied evaluation measures.

Measure	Description	Calculation
Specificity	*Measures the proportion of actual negatives that are correctly identified (also called the true negative rate)*	$Specificity=\frac{TN}{N}=\frac{TN}{TN+FP}$
Precision	*Precision is basically a ratio of the total detection*	$Precision=\frac{TP}{TP+FP}$
Recall	*Measures the proportion of actual positives that are correctly identified*	$Recall=\frac{TP}{P}=\frac{TP}{TP+FN}$
Accuracy	*Accuracy in the general statistical sense denotes the closeness of computations or estimates to the exact or true values*.	$Accuracy=\frac{TP+TN}{P+N}$
F1	*F1 score (also F-score or F-measure) is a measure of a test’s accuracy*. *It considers both the precision and the recall of the test to compute the score*	$F1=\frac{2TP}{2TP+FP+FN}$
MCC	*It is used as a measure of the quality of binary (two-class) classifications*	$MCC=\frac{TP\times TN-FP\times FN}{\sqrt{\left(TP+FP)(TP+FN)(TN+FP)(TN+FN\right)}}$
FDR	*It is a method of conceptualizing the rate of type I errors in null hypothesis testing when conducting multiple comparisons*	$FDR=\frac{FP}{FP+TP}$
NMI	*NMI is a measure of the mutual dependence between the two variables*. *More specifically*, *it quantifies the "amount of information" obtained about one random variable through observing the other random variable*	$NMI(Y,C)=\frac{2\times I(Y;C)}{\left[H(Y)+H(C)\right]}$

## Results and discussion

### Networks properties

In this subsection, we briefly describe the properties of networks employed in the two stages of the workflow. Topological properties of datasets, were reported in Table 2. As mentioned before, the real social multilayer was obtained and the two synthetic multilayers were simulated from single-layer modular graphs. In the simulation of a multilayer network from its single-layer modular graph, to be fair in generation of layers (S1 Appendix) edge-selection-probability parameter was set to 0.5. Manually, the layer count value was set to 5, but it was possible to set any other value (generate any number of layers). It should be noted, all layers are undirected and unweighted graphs.

**Table 2:** Topological properties of multilayer networks employed in the study.

Dataset	Number of nodes	Number of edges	Number of modules	Number of layers
Multilayer1	61	1240	8	5
Multilayer2	100	778	5	5
Multilayer3	100	1526	5	5
COAD	11044	120825	-	2

At stage B, The obtained dataset from the TCGA includes total RNAseq expression data for 60483 coding/non-coding RNAs in 49 samples that consisted of samples collected from patients with colon cancer. Then, we carried out gene filtering as described in the “Materials and Methods” section. The cleaned data include the expression count value for 14515 genes in 49 samples. The co-expression layer type was set to ‘unsigned’ and it was excerpted for the most correlated genes (correlation threshold was set to 0.8 to consider only 20% of high correlations [66]). Nevertheless, in the physical binding layer, all protein interactions for the same nodes of the first layer were selected. The generated co-expression layer comprises 5993 nodes and 75121 edges. On the other side, the physical interaction layer was directly generated from source datasets. The physical interaction layer (PPI) has 12751 nodes and 135712 edges.

### PLCDM comparison

In order to verify the PLCDM, in an iterating process every node in the ground-truth communities of the three evaluation datasets, was considered as the seed node and the output community of that seed were computed (predicted) through PLCDM, ML-LCD and Seed-Set-Expansion methods. Then, the three predicted communities are compared with the original ground-truth community based on different evaluation metrics.

In the PLCDM execution, the Jump probability was calculated using trial and error procedure. We set this value to 0.5 so that the walker could reach to most nodes (in some cases, some nodes are isolated and therefore unreachable) and score nodes around the seed with higher probability.

**Fig 3:**
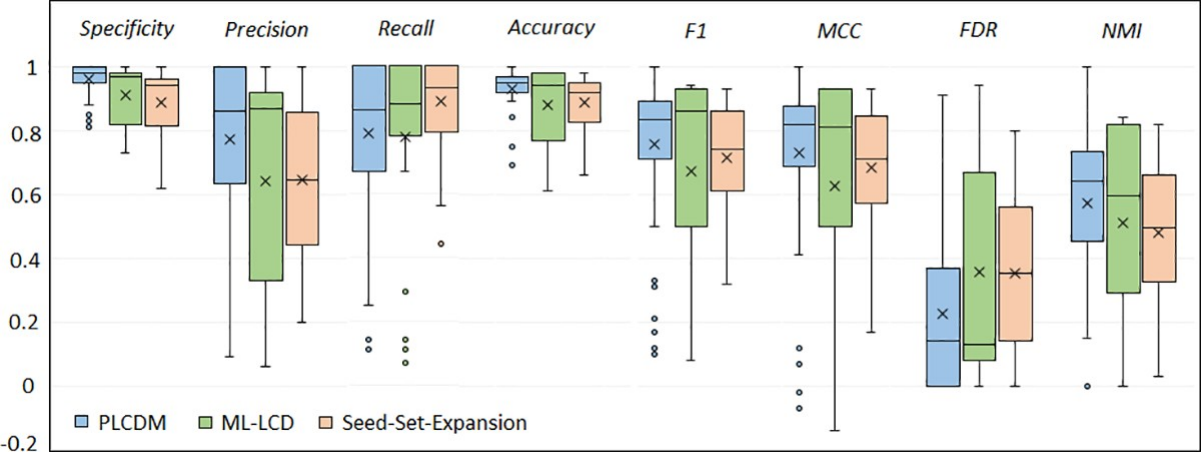
PLCDM performance in comparison with other methods ML-LCD and seed-set-expansion. Using Multilayer3 dataset (real data for social connections), in the predefined ground-truth communities, every node was selected as the seed node, and using three local community detection methods (PLCDM, ML-LCD, and Seed-Set-Expansion) result community was predicted for that selected seed. Then, the predicted community of each method was compared to the original ground-truth community and evaluated using eight metrics. As demonstrated, except Recall, in all measures PLCDM has better results. Notably, the false discovery rate of PLCDM is smaller than the other two methods. Every boxplot demonstrates the values gained by a method in this iterative process. Therefore their inter-quartile range and their average are comparable.

The results of this evaluation is illustrated in Fig 3. On the Multilayer1, in all extents of evaluation measures except Recall, PLCDM outperforms the others (Table 3). Nevertheless, in the second dataset (Multilayer2), ML-LCD’s performance is superior in five measures and PLCDM gains excellence in terms of FDR, Specificity, and Precision (Table 4). For the Multilayer3, as revealed in Table 5, PLCDM performs much better than ML-LCD and Seed-Set-Expansion methods.

**Table 3:** Evaluating PLCDM the Multilayer1 data.

	Specificity	Precision	Recall	Accuracy	F1	MCC	FDR	NMI
PLCDM	**0.97**	**0.81**	0.7	**0.92**	**0.72**	**0.7**	**0.19**	**0.53**
ML-LCD	0.91	0.64	0.78	0.88	0.67	0.63	0.36	0.51
Seed-set- expansion	0.89	0.64	**0.89**	0.89	0.71	0.68	0.36	0.48

**Table 4:** Assessing PLCDM using the Multilayer2 data.

	Specificity	Precision	Recall	Accuracy	F1	MCC	FDR	NMI
PLCDM	**0.99**	**0.9**	0.45	0.87	0.6	0.58	**0.1**	0.34
ML-LCD	0.93	0.78	**0.78**	**0.9**	**0.78**	**0.72**	0.22	**0.67**
Seed-set- expansion	0.96	0.75	0.43	0.85	0.54	0.49	0.25	0.22

**Table 5:** Comparison of PLCDM with other methods based on the Multilayer3 data.

	Specificity	Precision	Recall	Accuracy	F1	MCC	FDR	NMI
PLCDM	**1**	**0.97**	**0.92**	**0.98**	**0.94**	**0.94**	**0.03**	**0.85**
ML-LCD	0.97	0.86	0.91	0.96	0.88	0.86	0.14	0.84
Seed-set- expansion	0.88	0.58	**0.92**	0.89	0.71	0.67	0.42	0.42

### Local vs. global community detection

**Fig 4:**
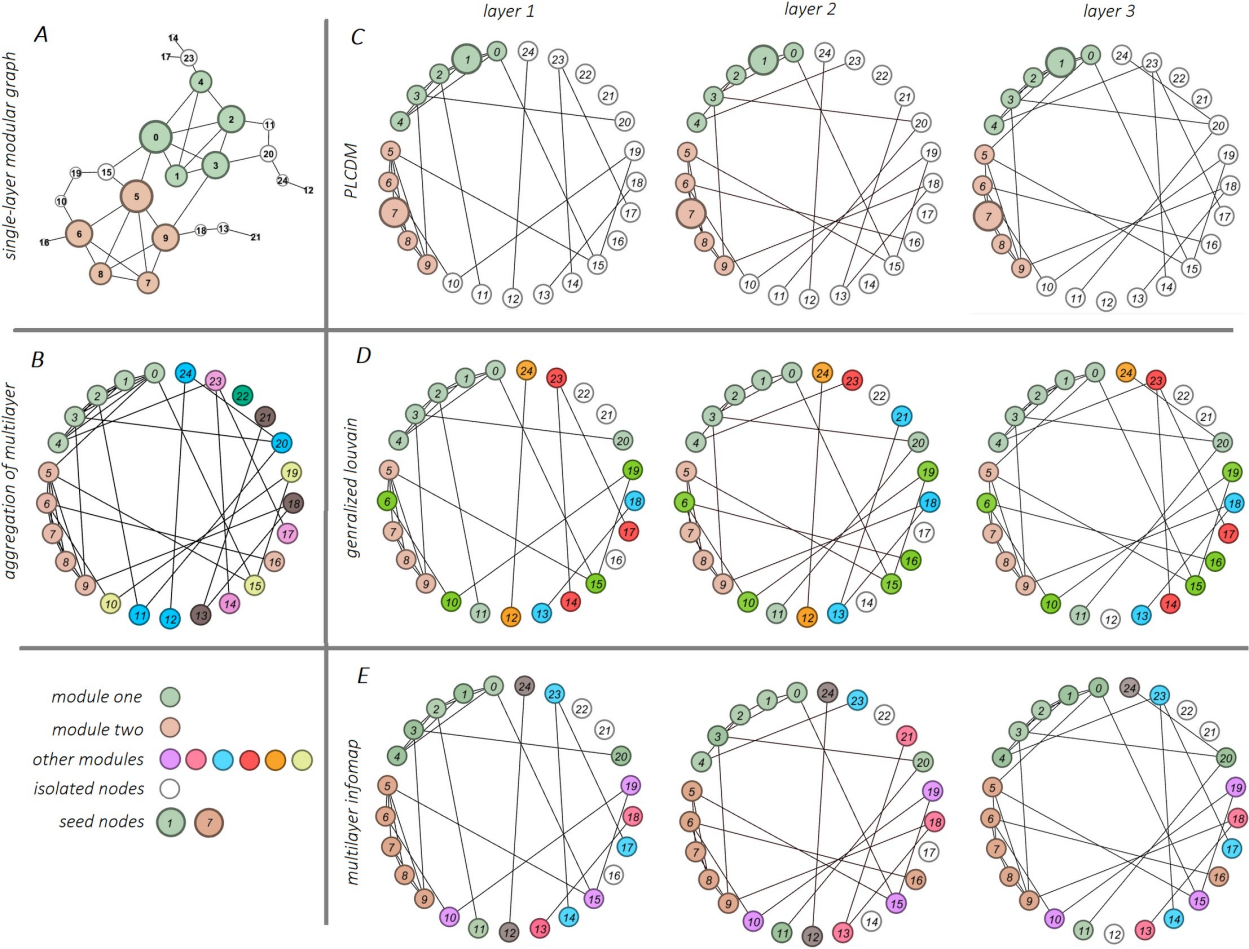
Local community detection. **Global vs.** Here, a synthetic multilayer network of containing 25 nodes in three layers and two modules of size five ({0,1,2,3,4} *and* {5,6,7,8,9}), as gold-standard modules, were used to compare global versus local community detection methods. A) The original single-layer modular graph and its ground-truth communities. B) The aggregated network of multilayer in which communities were unfolded by the main Lovain algorithm. C) The PLCDM method were applied on seed nodes {1, 7} in two separate executions. For every seed node, the detected module is matched with the ground-truth one. D) The global method, generalized Louvain (gLouvain), applied to the network, and six modules of different sizes were extracted. E) The multilayer Infomap, applied to the network and global communities were disclosed.

The PLCDM is a local community detection scheme and its objective is to the discover a single local community that the predefined seed node belongs to it. However, the global community detection methods are used to acquire any community structure in the network. Here, we make a comparison between local and global methods, which is illustrated in Fig 4. For this purpose, a typical synthetic multilayer network was employed that was created by the “modular graph generation” and “multilayer simulation from single-layer graph” algorithms, having 25 nodes and two modules of size five in three layers. The original single-layer modular graph is illustrated in Fig 4A. First of all, communities of the aggregated single-layer graph, unfolded by Louvain [28] algorithm, was displayed in Fig 4B. Secondly, the three methods PLCDM (local, Fig 4C), generalized Louvain (gLouvain) [67] (global, Fig 4D), and multilayer Infomap [44] (global, Fig 4E) were applied on the multilayer network. For the PLCDM, nodes 1 and 7 were randomly specified as initial seed nodes, in two separate executions. However, in the other two methods, communities other than the two communities in question are also extracted. It is noticeable that the PLCDM detects both communities in a precise way.

As pointed previously, we set the walker to move from edges present in most layers to focus on strong edges. Here, in a separate step, to show the effect of the edges that are present in most layers and to observe the difference between a biased random walk on the multilayer network and its aggregated network, Table 6 is presented.

**Table 6:** Biased random walk on a typical multilayer network versus its aggregated network.

Node	Biased RW on ML	Biased RW on aggregated of ML
0	0.29	0.37
1	1.00	1.00
2	0.46	0.45
3	0.10	0.15
4	0.36	0.20
5	0.02	0.01
6	0.00	0.00
7	0.01	0.00
8	0.00	0.00
9	0.00	0.00
10	0.00	0.00
11	0.01	0.00
12	0.00	0.00
13	0.00	0.00
14	0.00	0.00
15	0.02	0.01
16	0.00	0.00
17	0.01	0.00
18	0.00	0.00
19	0.00	0.00
20	0.03	0.00
21	0.00	0.00
22	0.00	0.00

### Application of PLCDM on COAD-specific multilayer network

**Fig 5:**
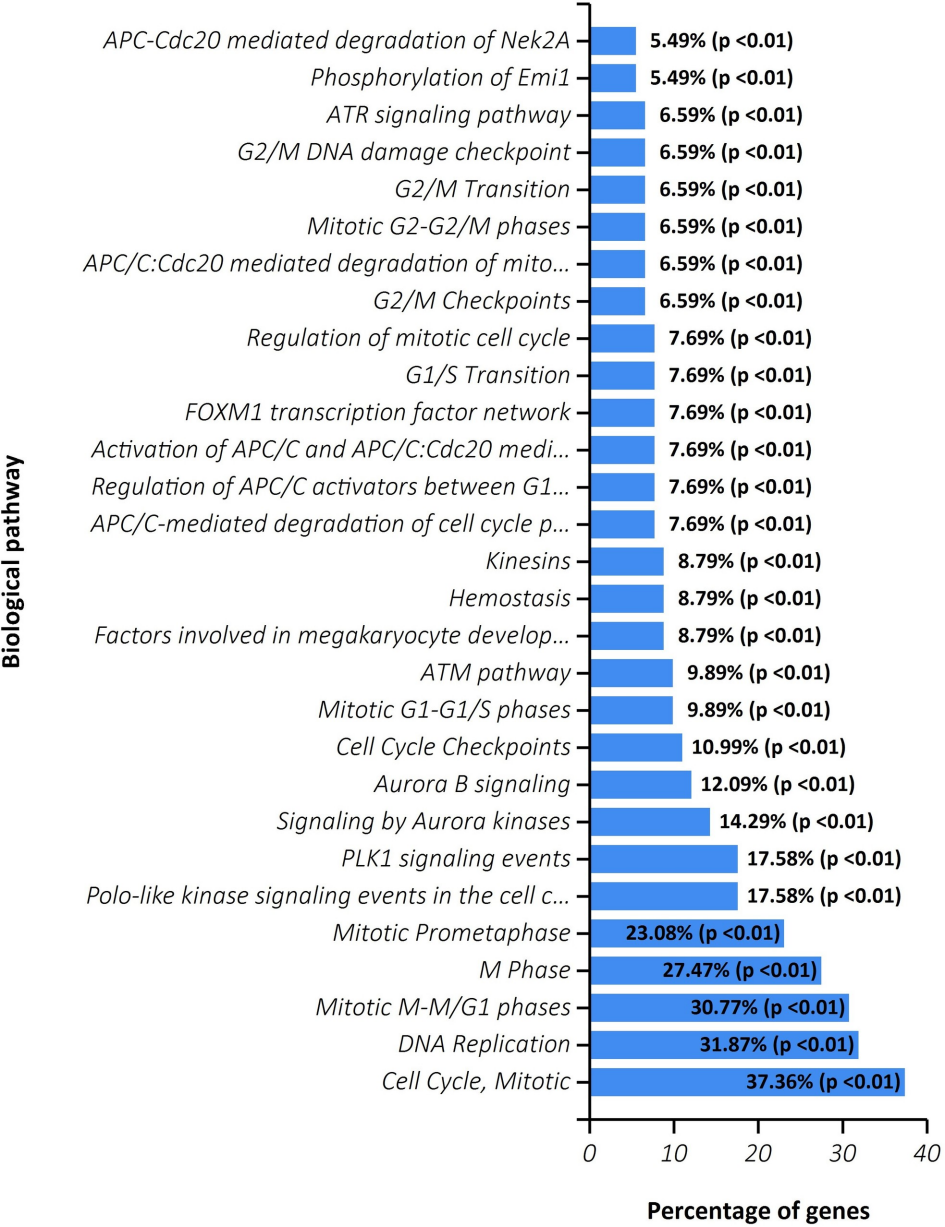
Pathway enrichment result of explored module around the seed gene HMMR.

Once the assessment process was achieved, as a case study, we applied the PLCDM on the colon cancer network as we clarified formerly. We ran the method by manually seeding it with *HMMR* as a known gene involved in cancerous signaling pathways. The PLCDM-explored community around the *HMMR* comprises 97 genes that computationally are similar in function and action. To confirm this finding, we benefited ToppFun enrichment portal of ToppGene [68] and FunRich [69] utilities and studied the role of discovered biomolecules in tumor-associated pathways and biological processes. The pathway enrichment results of the output module are shown in Fig 5. However, the detailed information of gene ontology (biological processes, molecular function, and cellular component), pathways, protein domain, and expression site are accessible in S2 Appendix. The outcomes suggest that the objects exploited in the yielded module are related to pathways involved in cell growth and proliferation, and carcinogenesis of the colon, such as *Cell Cycle*, *DNA Replication*, *APC/C-mediated degradation of cell cycle proteins*, and *Mitotic M-M/G1 phases*. As reported by the ToppFun, this module is correlated with the *Colorectal Neoplasms* significantly (p-value = 3.433E-5).

The *APC* produces a protein that acts in the *Wnt signaling pathway* with a tumor-suppressing function. It has roles in other biological processes, such as *cell migration and adhesion*, *transcriptional activation*, and *apoptosis*. Defects in the APC lead to *familial adenomatous polyposis (FAP)* that commonly moves toward malignancy. Mutations in this gene have been shown to occur in most colorectal cancers [70]. Additionally, it is closely associated with *cell division*, which is an important aspect of cancer cells.

The extracted module is involved in the *FOXM1 transcription factor network*. The *FOXM1* gene encodes a transcriptional activator protein that is involved in cell proliferation. This protein is phosphorylated in the *M phase* and controls numerous genes in the cell cycle (e.g., cyclin B1 and cyclin D1). For the *FOXM1*, several transcript variants coding isoforms have been found (https://www.genecards.org/).

**Fig 6:**
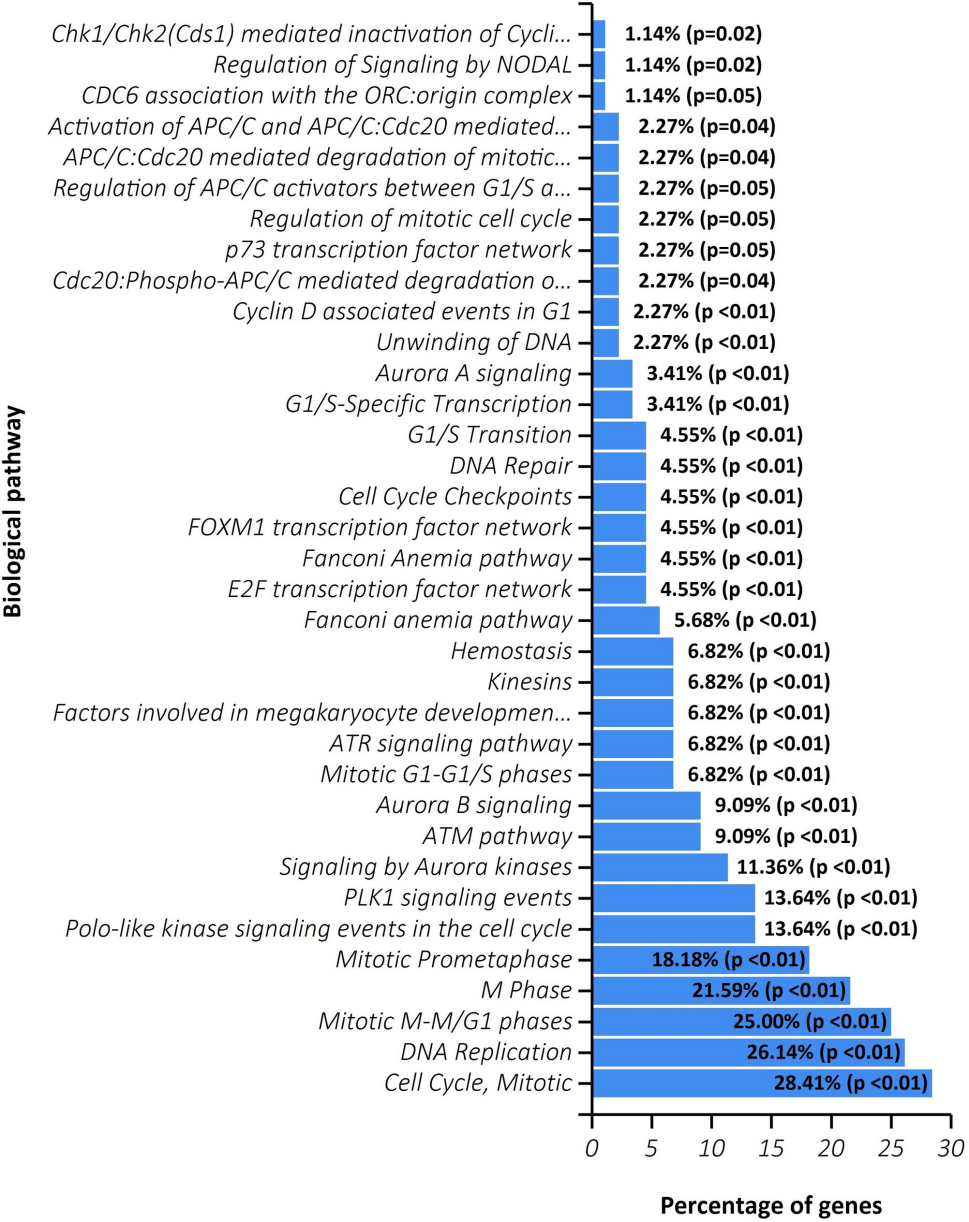
Pathway enrichment result of explored module around the seed gene ECT2.

Next, in another parallel execution, the method was applied with the seed *ECT2* that is a colorectal cancer-associated gene. This time, the extracted module comprises 101 genes. The functional enrichment results demonstrate that the gene-set is associated with *apoptosis*, *cell proliferation*, *cell cycle*, *DNA repair*, and *DNA replication* significantly (Fig 6). All the stated pathways are essential for carcinogenesis and the rapid development of tumor cells. Cyclin D plays role in controlling cell cycle development. The cyclin D synthesis is originated during G1 and excites the G1/S phase transition. Li et al. [71] previously mentioned on Prognostic Significance of Cyclin D1 Expression in Colorectal Cancer. Here, the genes in the output module are correlated with the *Cyclin D-associated events in G1*. For more information about these results, see S3 Appendix.

## Conclusion

Communities are the core and functional parts of networks that contain objects similar in form or behavior. In this way, numerous community detection algorithms were proposed to reveal modules in different network types, such as multilayer networks. In real-world applications, sometimes, finding communities in a network is not as important as extracting the superior community of a solitary preexposed node. As a biological case, in cancer applications, when an oncogene is known experimentally, finding its related component is more important than the discovery of every module in the whole system (that may be non-correlated to cancer). Herein, we propose a new seed-centric local community detection method for the multilayer environment that is based on information cascade theory. In this method, by propagating information from the predefined seed, nodes in the neighborhood of the seed are selected based on the information proportion that they obtained. To confirm the algorithm, various multilayer datasets with predefined ground-truth communities were employed. The results designate that PLCDM does well against earlier approaches with regard to evaluation measures. Finally, we applied the method on reconstructed colon adenocarcinoma multilayer networks and examined the outcome modules in terms of functional enrichment and GO annotations. Our assessments disclose that the local modules are correlated with pathways and biological processes involved in colon cancer.

## Supporting information

S1 Appendix Description of the two supplementary algorithms: The *modular graph generation* and the *multilayer simulation*. (DOCX)

S2 Appendix Functional enrichment results for the local module exploited for the seed gene *HMMR*. (XLSX)

S3 Appendix Functional enrichment results for the local module exploited for the seed gene *ECT2*. (XLSX)
