# Supplementary material for: A propagation-based seed-centric local community detection for multilayer environment: The case study of colon adenocarcinoma
Source: PLoS One. 2021 Aug 9;16(8):e0255718. doi: 10.1371/journal.pone.0255718 (PMC8351981; doi:10.1371/journal.pone.0255718)
Supplement: S1 Appendix — (DOCX) [file pone.0255718.s001.docx]

A propagation-based seed-centric local community detection for multilayer environment: the case study of colon adenocarcinoma

(A novel multilayer local community detection for biological modeling)

Ehsan Pournoor­^1^, Zaynab Mousavian^2^, Abbas Nowzari-Dalini^2^, Ali Masoudi-Nejad^1*^

^1^ Laboratory of Systems Biology and Bioinformatics (LBB), Institute of Biochemistry and Biophysics, University of Tehran, Tehran, Iran

^2^ School of Mathematics, Statistics, and Computer Science, College of Science, University of Tehran, Tehran, Iran

Description of the two Supplementary algorithms: The *modular graph generation* and the *multilayer simulation*

# Modular graph generation and multilayer simulation algorithms

Because of the limitations in ground-truth multilayer networks with identified communities, to create an artificial ground-truth dataset we proposed two algorithms for modular graph generation and multilayer simulation. When the modular graph generated, its gold-standard modules are specified. After simulating a multilayer network from the modular graph, the goal is to discover modules in the multilayer network that are very similar to the gold-standard communities pre-specified in the original single-layer graph.

# Modular graph generation

In the proposed algorithm of modular graph generation (Alg. 1), five parameters were specified: (1) number of nodes in the generated graph (node_num), (2) number of modules or communities in the graph (mod_num), (3) size of each module (mod_size), (4) probability of edge existance between two nodes inside a community (in_prob) and (5) the probability of edge existence between nodes outside of a community (out_prob). The algorithm simply, first, assigns mod_num modules of size mod_size. Then adds edges between every two nodes in the graph based on the criteria that two nodes are in the same module or not. In our test, the parameter in_prob was set to 0.8 and out_prob to 0.1. A sample generated modular graph using the proposed algorithm is depicted in Fig. 1.

| ***Algorithm 1. Modular graph generation*** |
| --- |
| 1. **Input:** *node_num, mod_num, mod_size, in_prob, out_prob* 2. **Output:** *graph* 3. create empty *graph* with *node_num* nodes 4. select *mod_num* modules of size *mod_size* from *node_num* nodes 5. for every nodes *a*, *b* in nodes: 6. if (*a* == *b*) continue 7. if (*a* and *b* are in the same module): 8. add edge (a, *b*) with probability of *in_prob* 9. else: 10. add edge (*a*, *b*) with probability of *out_prob* 11. return *graph* |
|  |


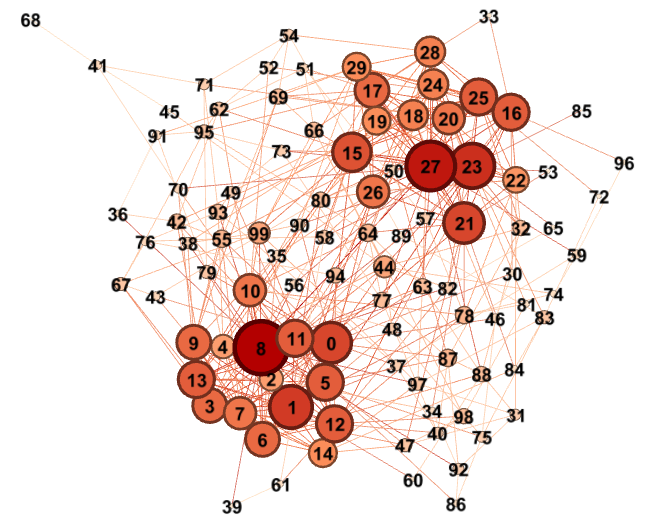


Figure S1. An artificial modular graph generated using Algorithm 1.

# Multilayer simulation

Multilayer simulation is used to provide a multilayer network form a single-layer graph. We employed this algorithm (Alg. 2) to prepare multilayer networks from the two artificial modular graphs mentioned in the manuscript. The parameters in the algorithm pseudocode are a single-layer graph (single_layer_graph), the number of layers (num_layers), and edge selection probability (edge_sel_prob). In our experiments, we set the edge_sel_prob to 0.5. A sample multilayer simulation for the graph illustrated in Fig. 1 is shown in Fig. 2.

| ***Algorithm 2. Multilayer simulation*** |
| --- |
| 1. **Input:** *single_layer_graph, num_layers, edge_sel_prob* 2. **Output:** *multilayer* 3. *multilayer* = a list of *num_layers* empty graphs 4. add all nodes of *single_layer_graph* to every layer *l* in *multilayer* 5. for every edge *e* in *single_layer_graph:* 6. for every layer *l* in *multilayer:* 7. Add *e* to *l* with probability of *edge_sel_prob* 8. return *multilayer* |
|  |


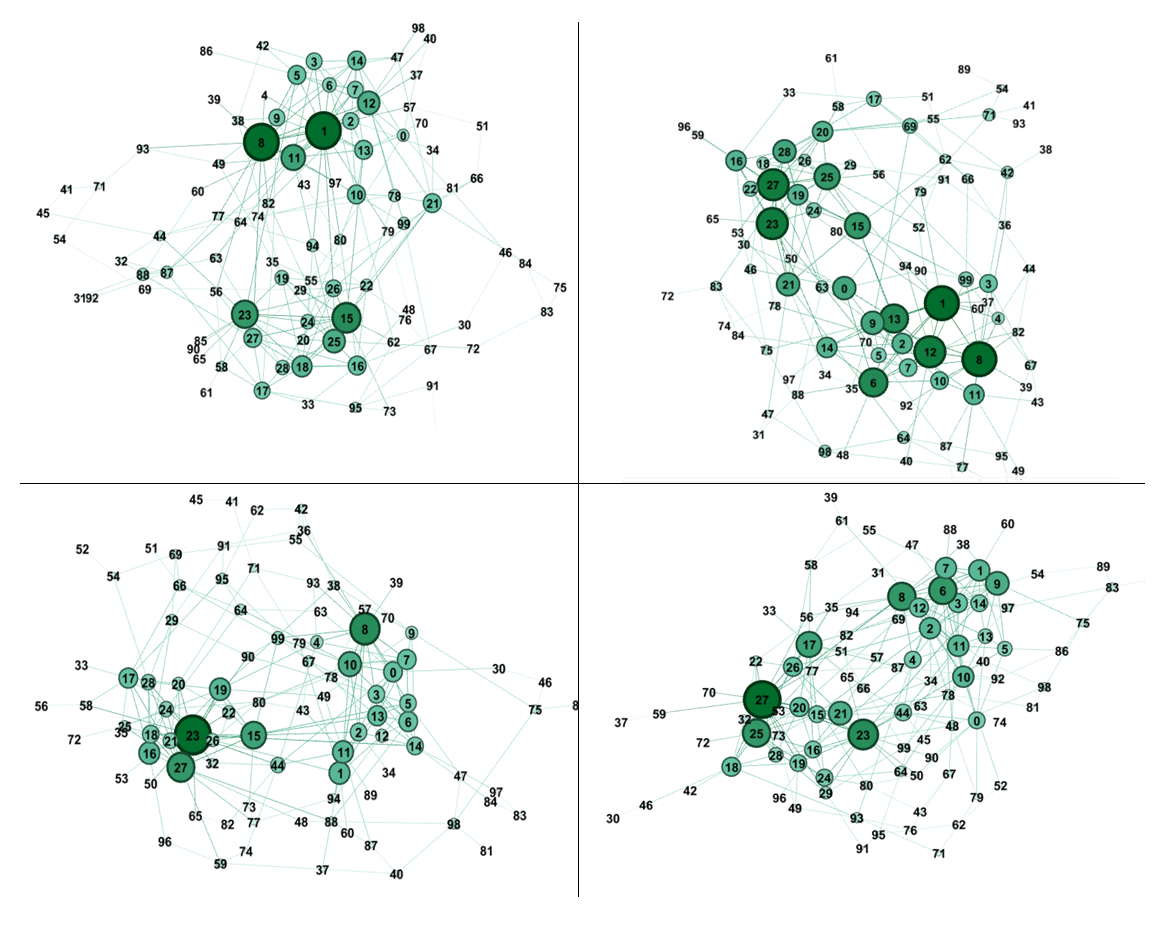


Figure S2. A four-layer multilayer network simulated from a single-layer graph demonstrated in Fig. S1.
